# Steganography in IoT: Information Hiding with APDS-9960 Proximity and Gestures Sensor

**DOI:** 10.3390/s22072612

**Published:** 2022-03-29

**Authors:** Katarzyna Koptyra, Marek R. Ogiela

**Affiliations:** Cryptography and Cognitive Informatics Laboratory, AGH University of Science and Technology, 30-059 Krakow, Poland; kkoptyra@agh.edu.pl

**Keywords:** stegaography, IoT, gestures, sensor, APDS-9960

## Abstract

This article describes a steganographic system for IoT based on an APDS-9960 gesture sensor. The sensor is used in two modes: as a trigger or data input. In trigger mode, gestures control when to start and finish the embedding process; then, the data come from an external source or are pre-existing. In data input mode, the data to embed come directly from the sensor that may detect gestures or RGB color. The secrets are embedded in time-lapse photographs, which are later converted to videos. Selected hardware and steganographic methods allowed for smooth operation in the IoT environment. The system may cooperate with a digital camera and other sensors.

## 1. Introduction

Internet of Things (IoT) is a fast growing sector that has made great progress, but still faces many challenges. The main achievements in this area are in efficiency, energy-saving and new functions. They find application in smart buildings, the e-health industry, big data [1], remote monitoring [2] and many more. The requirements for such systems are usually high: they are supposed to fulfill complicated tasks in near real time. These may be object detection and recognition, feature discovery, signal classification or data analysis. Some solutions evolve towards machine learning and other intelligent algorithms, and others focus on optimization or cost reduction. For example, energy consumption may be reduced with a specialized algorithm [3] or better routing [4]. The intelligent use of sensors facilitates operation in difficult conditions [2] to reduce manual work. As a result, highly specialized devices are available at a relatively low price. This, however, comes at the expense of security, which is treated as an unnecessary expenditure.

Security threats in IoT are studied by the OWASP foundation [5]. They analyze the main vulnerabilities and attack surface areas. The goal of the project is to “help Developers, Manufacturers, Enterprises, and Consumers to make better decisions regarding the creation and use of IoT systems”. Among the most serious threats, we may find weak, guessable or hardcoded passwords; a lack of a secure update mechanism; insufficient privacy protection; insecure data transfer and storage, etc. These problems cannot be easily solved if the software cannot be updated. Replacing hardware is also not an option because of a lack of secure components (the old device is replaced by a new device that is also insecure). The one approach to address this problem is to build the whole system from scratch, with selected hardware and software created by hand. Sometimes this may be the best solution, but is it expensive and time-consuming, so another possibility is to add a new layer of protection to the system. Then, only a few new elements are added and a small portion of code is written.

Data protection in IoT systems may be realized in multiple ways. The secrets may be encrypted, hidden, separated from public networks and so forth. This paper focuses on the technique of data concealment, which is called steganography. Its main goal is to hide secret data inside an inconspicuous medium that plays the role of cover. Steganographic techniques use various media, such as images [6], for which, new improvements are proposed [7]. Such algorithms in IoT should consider not only undetectability, but also efficiency. The research in this field [8] showed that the efficiency of IoT systems is enough for steganographic purposes. Therefore, the main goal of this article is to present a new method of information hiding. Its novelty lies in using a gesture detection sensor that works in two modes.

## 2. Materials and Methods

### 2.1. Hardware

**Figure 1:**
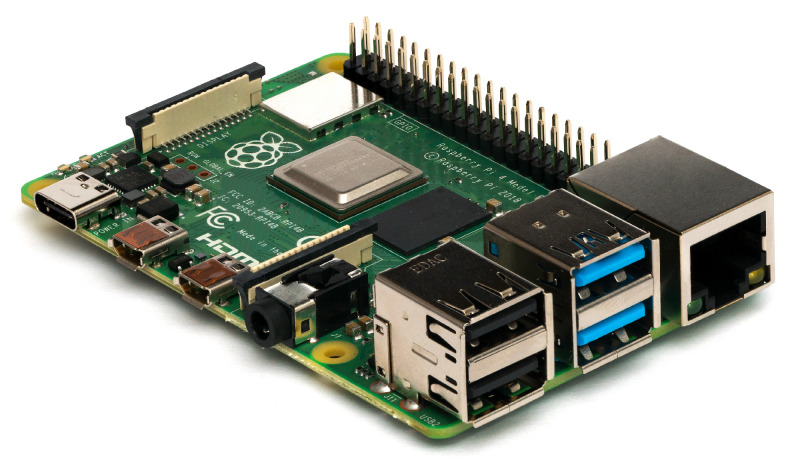
Raspberry Pi 4 Model B (Laserlicht/Wikimedia Commons/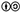).

The project was made on Raspberry Pi 4 Model B, which is a tiny, credit-card-sized computer (Figure 1), usually used as a robot brain, smart home hub, media center, factory controller, etc. [9]. The chosen version has 8 GB of RAM. It is equipped with a 1.5 GHz 64-bit quad core ARM Cortex-A72 processor, two micro HDMI ports, two USB 3.0 ports, two USB 2.0 ports, 802.11ac Wi-Fi, Bluetooth 5 and gigabit Ethernet. The board is powered via a USB-C port and requires a 5 V supply.

**Figure 2:**
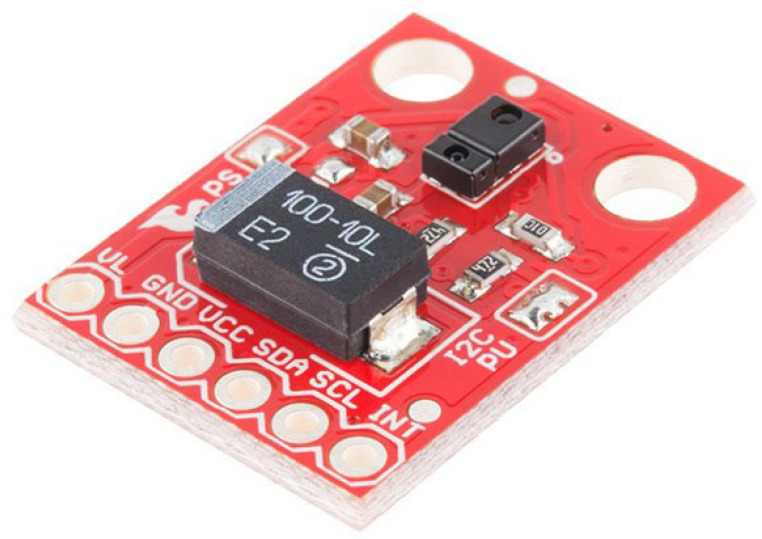
APDS-9960 gesture sensor (SparkFun Electronics https://sparkfun.com/ (accessed on 21 March 2022)).

The core element of our steganographic system is APDS-9960 (Figure 2). This is a sensor that measures ambient light, RGB color and proximity, and also detects gestures. Therefore, a user may control a device by swiping hands over the sensor. The gesture engine is able to sense not only simple UP-DOWN-RIGHT-LEFT swipes, but also a wide range of more complex gestures [10]. The sensor may be found in real-life devices; for example, Samsung Galaxy S5. It is powered by 3.3 V and uses inter-integrated circuit (I2C) communication protocol.

An additional device used in our project is OV5647 camera with native resolution of 5 megapixels [11]. The camera is capable of 2592 × 1944 pixel static images and also supports 1080p30, 720p60 and 640 × 480p60/90 video. It uses the camera serial interface (CSI)—flat ribbon cable, which is dedicated for cameras. The size of board is 25×24 mm and the angle of view is 54×41 degrees. The camera can be controlled programmatically.

**Figure 3:**
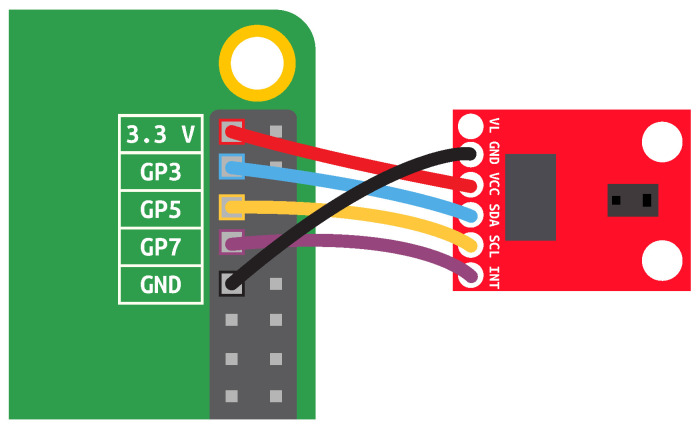
Hardware setup.

The hardware setup is presented in Figure 3. Voltage common collector (VCC) should be connected to a power pin. Rasbperry Pi has four power pins (two 3.3 V and two 5 V) used as a source to power external peripherals. The obvious choice is to use 3.3 V pin. Ground (GND) of sensor is connected to GND of the board. Serial data (SDA) and Serial clock (SCL) should be connected to I2C pins of Raspberry Pi: SDA pins are 3 and 27; SCL pins are 5 and 28. We chose nearby pins 3 and 5. Interrupt (INT) of the sensor is connected to general purpose input/output; for example, pin 7. If camera is used, it is connected to CSI (not depicted in the scheme, but visible in Figure 1 as a long flat socket next to HDMI).

### 2.2. Configuration

The camera interface and I2C should first be enabled in raspi-config. This allows for serial communication and taking photos. Pin 7 needs to be in input mode to read data from the sensor. Additionally, required Python libraries should be installed: apds9960 (for the gesture sensor) and pyexiv2 (for embedding and extracting data). Other components, such as raspistill application used for time-lapse photography, are preinstalled on Raspbian.

### 2.3. Methods

The steganographic scheme works in an environment in which there is a series of images in JPEG format that are later combined into a video; for example, in time-lapse photography. The images may be captured with digital camera or come from another source.

Data hiding is controlled by gestures. The sensor may operate in two modes: as a trigger or data input. In trigger mode, some gestures invoke embedding process, which is continued until finishing gesture is encountered. The data are pre-existing or come from external input. In data input mode, the data come directly from the sensor, but, before embedding, they are first encoded with chosen coding system. If images pre-exist, the data are hidden as they appear; when images are captured in real time, the data are queued and wait until new covers are available.

Embedding algorithm uses features of JPEG format specification to hide data. In JPEG, the header of a file consists of segments, each of which starts with a pair of bytes called marker. The first byte of a pair is always 255, the second may vary depending on segment type. For example, comment (COM) is identified with marker 254. The structure of a comment is not imposed. Usually, it contains information about quality, program used to create the image, etc. [12] In this study, secret data were concealed inside the COM marker. The reason for this choice is that metadata-based methods are independent of image content, so their speed is predictable, which is an important factor in IoT environment.

Secret recovery is triggered when the photos are finally combined to form a video. In that moment, the message is revealed. Formally, is it not necessary to create a video to recover the data. However, videos are set a specific time frame and limit the capacity (maximum amount of data possible to conceal). Therefore, the extracting process starts just before the video is generated.

### 2.4. Gesture Detection

The core element of presented system is gesture detection [13]. It is realized by apds9960 Python library, which can sense six types of gestures (also proximity, ambient light and RGB color). Left, right, up and down gestures are sensed when the user swipes the hand over the sensor in its range, which is 10–20 cm. On the other side, near and far are sensed when the hand is approaching the device or distancing from it. The detection uses interruptions when the falling edge is detected on INT pin.

### 2.5. Implementation Details

Time-lapse photographs are generated with the following command:

raspistill -t 600,000 -tl 2000 -o image%04d.jpg -w 1280 -h 720

This command takes one photo every two seconds and continues grabbing pictures for ten minutes. Generated images have resolution of 1280×720 and are named consecutively. In the experiments, shorter time frames (one, two and five minutes) were also attempted. The photos were later joined into a video with ffmpeg application.

The hiding algorithm uses comment section of JPEG header. It is based on the fact that each of the images have their own ID number. ID is created as a hash of a time when the photo was taken and some random number; for example, 1f8f8208fd27632661de715a5105882e eb93dca95421b5e97792b9c51de9e3d8. These identifiers have no special meaning, but are used to differentiate pictures. As a result of that, they may be slightly modified to conceal secret data.

In trigger mode, the embedding algorithm first computes XOR of all bytes of ID. The resulting byte should be equal to current message byte. If it is not, the algorithm randomly chooses one of ID bytes and changes it by XORing with message byte ⊕ previous XOR result. In this way, each image contains one byte of secret data.

Reading of gestures is realized with apds9960 library in Python. The concealment is triggered by near gesture; in other words, when the user put their hand (or other object) close to the sensor, the hiding procedure starts. This continues until the whole message is embedded. In trigger mode, it is possible to hide more than one message—each one should be started independently, at any moment after the previous one is finished.

Before embedding, the secret message is extended with null bytes at the end and with 255 bytes at the beginning. These bytes are used as indicators. Additionally, the message is encrypted (XORed with one byte key). This step is not necessary and has been added to make statistical analysis harder. The embedding process is presented in Algorithm 1 and assumes that all images are already available to show only the steganographic part. The whole scheme will be presented later. To extract the message, the user takes ID of image, calculates XOR of its bytes and repeats this for each image. Later resulting bytes are decrypted with one byte key. Finally, the user limits the message to areas indicated by 255 and 0 bytes. Algorithm 2 depicts these steps. Square brackets denote indexing, and colon inside means “elements reaching particular indices including both”.

**Figure 4:**
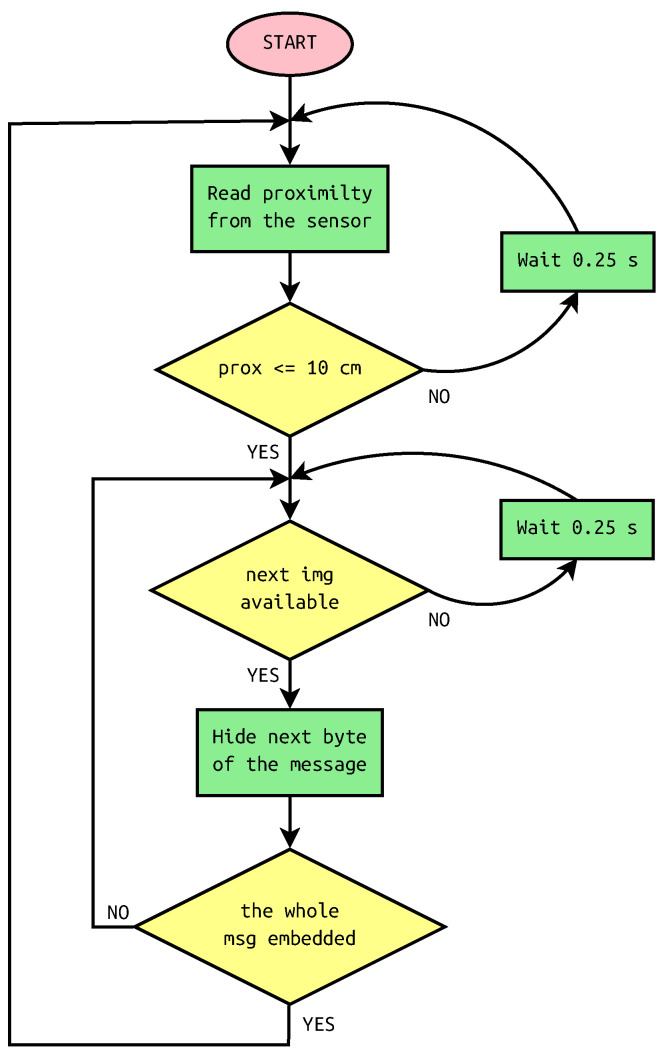
Flowchart of steganographic system with proximity sensor in trigger mode.

The scheme of steganographic system in trigger mode is depicted in Figure 4. As can be seen, the gestures are read every 0.25 s. This is the chart for multiple messages when the program is looped. Alternatively, for single message, the program returns after the whole message is embedded.

**Algorithm 1:** Embedding algorithm in trigger mode

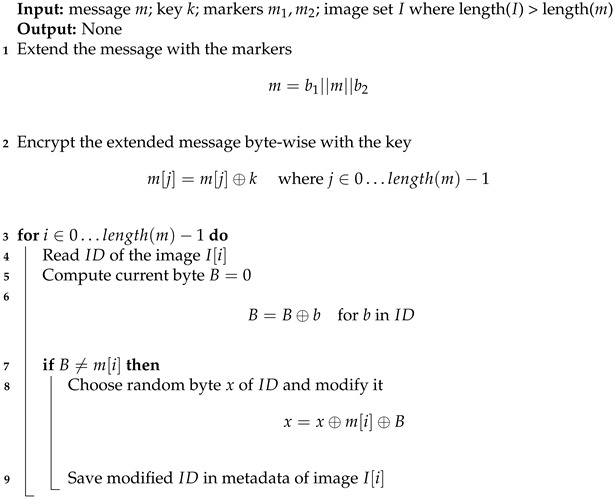



**Algorithm 2:** Extracting algorithm in trigger mode

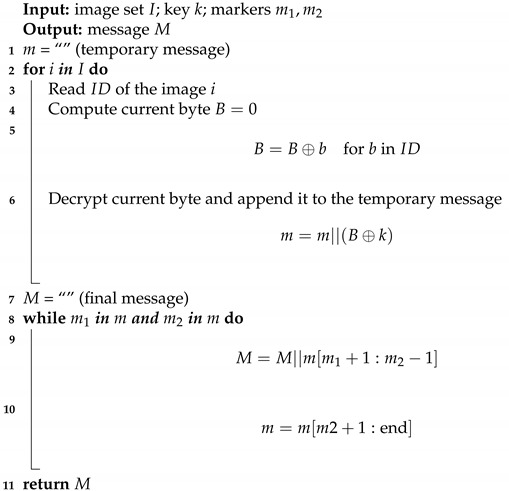



The implementation of data input mode is a little different. Each time a gesture is grabbed, one piece of information is hidden in a carrier. The sensor is able to differentiate six type of gestures, which are: left, right, up, down, near and far. Therefore, concealed message should be represented with a subset of possible gestures.

For example, the message can be written in Morse code, for which, we need to create encoding with three symbols. Let them be LEFT gesture for a dot (·), RIGHT gesture for a dash (−) and UP gesture for a gap (∣); any other gesture is ignored. Morse code is less efficient than ASCII encoding because one character needs approximately three symbols on average. However, we cannot directly encode all characters with only six types of gestures. The message “HAVE A NICE DAY” is encoded as ····∣·−∣···−∣·∣∣·−∣∣−·∣··∣−·−·∣·∣∣−··∣·−∣−·−−, which requires 45 gestures.

For consistency, the embedding algorithm is very similar to that in trigger mode. The one difference is that we need to be able to distinguish when the user performed significant gesture from other cases (gesture outside the chosen set or no gesture). This information is encoded in XOR of first three bytes of image ID—the even value means that secret data are embedded, the odd value means trash (random data). If LEFT, RIGHT and UP gestures are encoded as 0, 1 and 2, the following ID contains a dash: 760836bd30e8f738397add7e e9e24b3393e287d477c6665e43b2e20b495a9646. This method is depicted in Algorithm 3.

The extracting algorithm is run just before creating a video. It filters IDs of images to leave only meaningful data—those where their first three bytes are XORed with each other are even. Later, the algorithm computes XOR of each ID and decodes them to obtain Morse code symbols. Finally, the message is translated to ASCII and printed out. This method is presented in Algorithm 4.

**Figure 5:**
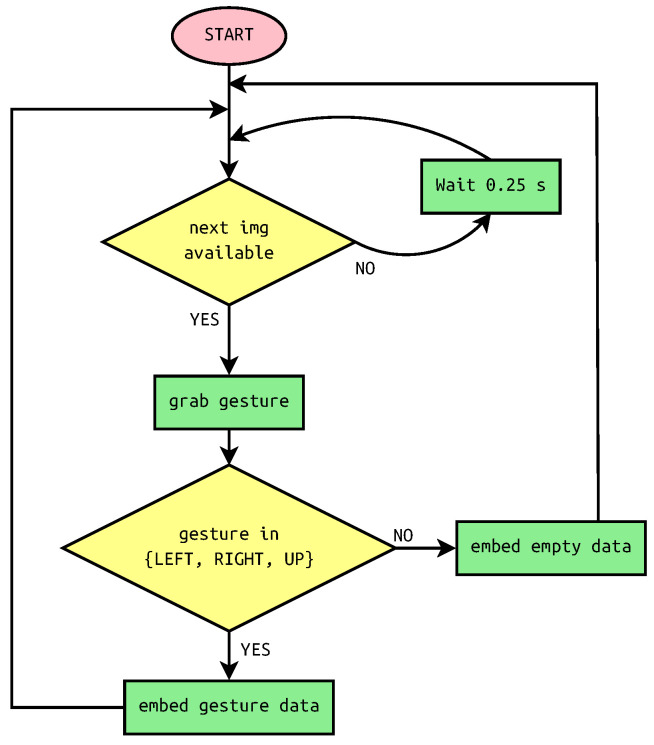
Flowchart of steganographic system with proximity sensor in data input mode.

The message from the example takes 90 s to be embedded when the gestures are performed one after another. This is because images are grabbed every 2 s. The system, however, allows for the user to perform gestures at any pace or with breaks. It is achieved by embedding trash data when no meaningful gesture is available. The flowchart of steganographic system in data input mode is presented in Figure 5.

**Algorithm 3:** Embedding algorithm in data input mode

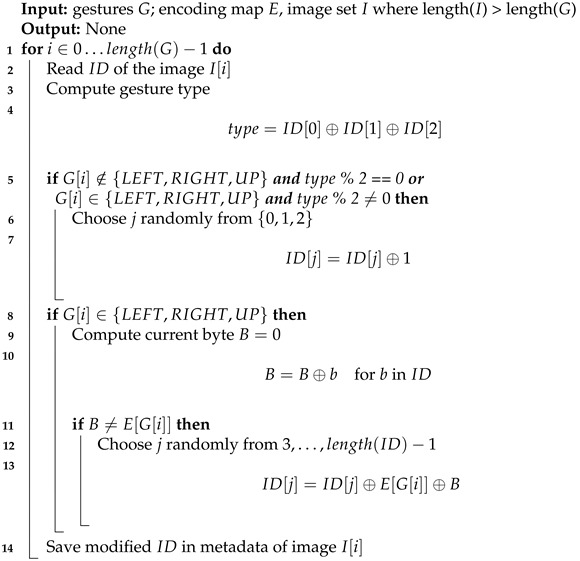



**Algorithm 4:** Extracting algorithm in data input mode

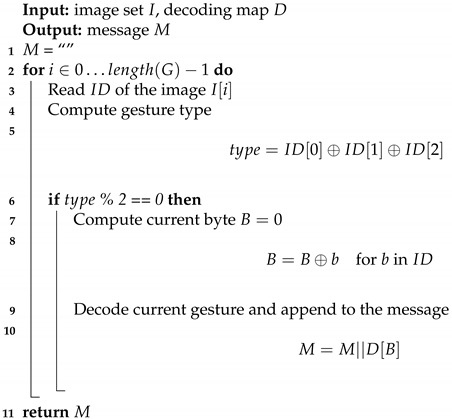



Experience shows that 0.25 s is enough time for grabbing a gesture because it is very hard to perform it faster. With such choice, gestures are not lost and the system runs smoothly without unnecessary delays.

In both modes, trigger and data input, steganographic application is written separately from time-lapse photography. There are two reasons for that. Firstly, capturing images with the camera and creating videos is identical for both modes, so code repetition is avoided. Secondly, the program may be easily incorporated into existing system without changing original application. This is important if steganography is used in a short period of time and later shredded to remove all traces.

### 2.6. Datasets

We did not use any external dataset. For real-time tests, we took pictures directly with the camera. For tests on pre-existing images we used previously taken photos. This is because the system has been designed to work with digital camera as a part of time-lapse photography project. Additionally, chosen steganographic algorithm uses metadata, so image content is not modified in any way, regardless of its origin.

## 3. Tests

The created system has been tested in both modes, for a single secret and multiple secrets. All secrets were English sentences without special characters. The messages have been successfully embedded and later extracted.

The analysis of containers confirmed that the hiding process does not change the content (pixels) of images. This is because the embedding algorithm modifies only metadata of the carrier file. Therefore, the one element that is changed is the identifier from the comment section of the file header. The identifier is formatted as a sequence of bytes without any special meaning, so its structure and length are identical with and without secret data. As a result, the file size remains unchanged.

Speed tests of the presented method showed that we may hide four bits per second. This is related to the reading frequency of the sensor and storing one byte in an image. The gestures are not read faster because of user experience tests, which revealed that trying to perform more than four gestures per second is not comfortable for the user and leads to carelessness and errors.

The experiments also revealed that creating a video takes exactly the same amount of time, regardless of whether secret data are present in carriers or not. The reason for this is because the embedding algorithm does not change the file size nor the pixel values.

## 4. Results

The study shows that the APDS-9960 sensor may be used in the IoT steganographic system, either to trigger the hiding process or to input data to conceal. In the former case, the secret is predefined, and in the latter, the user defines the message in the moment of performing gestures.

**Figure 6:**
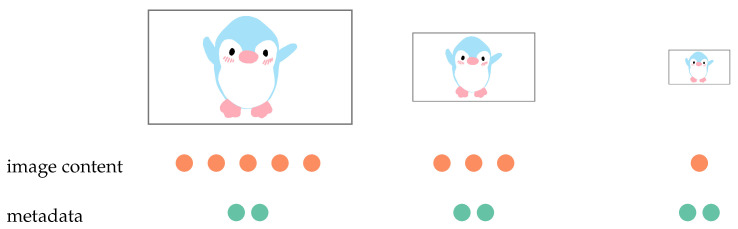
Relationship of image size to carrier capacity.

The chosen embedding method uses metadata of a JPEG format as a carrier for secret information. This has some consequences. The most important is that the capacity of the carrier is independent of the resolution of an image. The camera used in this research may work with a number of resolutions and the user may choose any of them, because the embedding method changes metadata only. Some steganographic algorithms hide a secret directly in pixels or the transform domain. Then, the capacity diminishes when the resolution is lower. On the other hand, algorithms that use metadata have a constant capacity. This relation is depicted in Figure 6. The number of dots shows how much information may be hidden in images with various resolutions using different approaches.

Moreover, the presented method has a constant amount of data embedded in a unit of time. It can conceal one byte per two seconds, which gives four bits per second, independently of current image size or content. In Internet of Things systems, a predictable speed of the algorithm is essential in order to ensure the responsibility of the system and to avoid delays. This is especially important when the steganographic part is added to existing application, so as not to distort time-lapse photography. Additionally, we had to to ensure that both systems ran in synchronization. That was relatively easy, as the frequency of grabbing gestures is much higher than taking photos.

It is possible to achieve a better capacity than one byte per image with a different encoding of the concealed message or a different embedding method. For example, pixel-based or frequency–domain techniques offer more space at the expense of a higher complexity. However, in the presented research, we prioritize the security and simplicity of the algorithm to enable working even with low-resource hardware. The messages were short so a low capacity was sufficient. This may be changed if needed.

It is also worth mentioning more about the security of the described method. The idea of using existing IDs of images gives an opportunity for perfect security. This is because the XOR operation produces an output that is indistinguishable from random data, provided that its second input is not predictable and is longer than the secret message. As IDs are generated with a hash function, the security relies on the proper selection of this function. We used sha256, which is suitable for the described application. The produced digests are much longer than a single byte, so the second condition of perfect security is also met. Besides, every image has its own ID, regardless of whether it contains secret data or not. As a result, an adversary is not able to find anomalies in files, nor to differentiate some images from others.

## 5. Discussion

Steganography in IoT is a promising topic and some solutions has already been proposed in this area. We may compare the presented method to others that also use a digital camera. The most frequent carriers are image [14,15] or video [16] files. These solutions may be classified as container modification algorithms that slightly change the content of container files. The method presented in this paper also changes the container, but it modifies the metadata of the file, not the content itself. Thanks to this approach, the algorithm has a predictable speed, which is essential in a restricted-resource environment.

Another advantage arises from the APDS-9960 gesture sensor. Usually, popular embedding techniques in IoT focus on efficiency, but are very similar to steganographic solutions in other areas. There is a predefined secret message that is later embedded in a carrier. The presented system may as well work in that way, but also has a possibility of operating in data input mode, in which, the message is passed directly to the sensor and concealed at the same time. This strength is important in IoT, because such settings are not always equipped with a convenient data input device.

There are some parts of the research that might be changed or implemented differently in order to open new possibilities and set future directions. Some of them are hardware-related and others are software-related.

The APDS-9960 sensor may detect not only gestures and proximity, but also colors. The device was tested with an RGB LED and seemed to work correctly. Therefore, in the future, it is possible to create a steganographic system controlled by light. Another opportunity is to test different sensors or new devices and to incorporate them in the system. They may work together with a digital camera and various microcontrollers.

From a software perspective, other carriers and algorithms can be used. Instead of hiding a message in metadata, we may decide to embed it directly in pixels or the transform domain, depending on the file format. For example, PNG is suitable for pixel-based methods [17] and JPEG is appropriate for transform-domain algorithms, such as F5 [18]. Alternatively, the data may be embedded in a video, not in photos. This solution offers a much higher capacity, but the embedding time is considerably longer and extracting is resource-consuming. Without the camera, it is possible to hide data in other types of containers [19].

All of these modifications are possible because the system has been written as an external application that was integrated into existing system of time-lapse photography.

To sum up, the steganographic system has been successfully deployed in the IoT environment. The chosen sensor worked well and was able to detect trigger events and capture gestures that encode data. Thanks to the efficiency of the algorithm, we achieved a low resource usage. Another important aspect is focusing on a high level of security. This topic is very important in IoT, as numerous devices suffer from many security problems [8]. The presented research confirms that steganographic systems can be used in practice for concealing secret data in IoT setups, and the results are promising in terms of efficiency and security.

## 6. Conclusions

Internet of Things systems are growing rapidly, but data protection is still at a low level. Steganography is one of the possible answers to security threats in the IoT environment. The APDS-9960 gestures sensor used in this research allows us to hide messages in an easy way and without attracting attention. The sensor is small and may be placed secretly; for example, under the desk. In this way, inputting data is not visible for external observers. The presented system is also user friendly, because gesture-based solutions are currently present in numerous applications and have become a natural way of communicating with devices.

This paper makes an effort to better protect secret data in IoT networks. It provides a steganographic system that is fast, convenient, secure and, at the same time, affordable for the average person. This small step in increasing data confidentiality may be an inspiration to create better systems and, hopefully, to produce more secure devices.

## Data Availability

Not applicable.
